# Healthy ageing in place: perspectives on age-friendliness in ‘local’ communities

**DOI:** 10.1186/s12889-025-26050-4

**Published:** 2025-12-23

**Authors:** Ragy Tadrous, Victoria J. Palmer, Jonathan R. Olsen, Martin Anderson, Benjamin P. Rigby, Meigan Thomson, Ruth Lewis, Kirstin Mitchell, Emily Long, Kathryn Skivington, Sharon A. Simpson

**Affiliations:** 1 School of Health and Wellbeing, University of Glasgow, Clarice Pears Building 90 Byres Road, Glasgow, G12 8TB UK; 2 Better Health and Care Hub, King’s College London, London, UK; 3 Institute for Social Science Research, The University of Queensland, Brisbane, Australia; 4 Population Health Sciences Institute, Newcastle University, Newcastle upon Tyne, UK

**Keywords:** Physical activity, Places, Older adults, Age-Friendliness, Localness, Place-based, Communities

## Abstract

**Background:**

Age-friendly communities are designed to support older adults in staying healthy, active, and engaged in society as they age. While the built environment plays an important role in their wellbeing, little is known about what shapes older adults’ views of community age-friendliness. This study explored how mid-to-older aged adults perceive community age-friendliness, and how these perceptions influence ageing in place.

**Methods:**

Semi-structured interviews (in-person/online) were conducted with 21 community-dwelling adults (55–75 years; mean age 65.0 ± 5.5) from two Scottish local authorities with high deprivation (September 2023 – March 2024). Guided by maps of their local area, barriers and facilitators to community age-friendliness were charted to the WHO Age-Friendly Cities, Place Standard and Ageing in Place frameworks, and perceptions of localness were explored.

**Results:**

Participants varied in physical activity, frailty, and loneliness levels, and experienced declining community age-friendliness. Physical barriers included neglected facilities, poorly accessible homes and spaces, and environmental hazards. Social barriers included declining access to social spaces due to rising costs, reduced hours and service closures. Service-related barriers included digital exclusion, ageism, safety concerns and reduced healthcare access. Perceptions of localness were shaped by (i) accessible and preferred transport options, with local areas shrinking as health declines; (ii) distance to essential services; and (iii) places where participants had social ties.

**Conclusions:**

The perceived deterioration of local places underpins a decline of community age-friendliness. To support older adults to age well, policymakers must consider the effects of declining mobility, access to- and closure -of essential facilities for older adults.

**Supplementary Information:**

The online version contains supplementary material available at 10.1186/s12889-025-26050-4.

## Introduction

The World Health Organization (WHO) describes an ageing global population as the most important medical and social demographic problem worldwide [1]. A significant increase in life expectancy and a growing population of older adults has been observed, with projections indicating that by 2030, approximately one-fifth (21.8%) of the United Kingdom’s population will be aged ≥ 65 years [2]. The concept of healthy ageing emphasises the importance of functional ability to support wellbeing in later life [3, 4]. However, as individuals age, their care needs typically escalate, with the percentage of people experiencing difficulties with activities of daily living increasing from 15% in those aged 65–69 years to approximately 33% by age 85 years [5].

Older people’s health is partially determined by their physical and social environments, such as their homes, neighbourhoods and communities, which can influence health directly or indirectly by shaping their opportunities, choices and behaviours [6]. The built environment can be defined as “human-made space(s) in which people live, work and recreate on a day-to-day basis” (p. 24) [7]. The built environment, and how older adults interact with it, is central to supporting healthy ageing, which considers the demands of the environment, individual needs, and the ageing process [8]. Inclusive physical and social environments can support older adults to pursue what matters to them, even with reduced physical capacity, by providing safe and accessible buildings, transport and walkable spaces [6]. Facilities like libraries, green spaces, fitness centres and community centres help foster community cohesion and social engagement [9]. Place-based approaches, including urban design and planning perspectives, can assist with understanding aspects of the built environment that can support healthy ageing [10].

The built environment is also important for ageing in place, which refers to older adults’ abilities to live safely and independently in their homes and communities [11]. Bigonnesse and Chaudhury [24] proposed an Ageing in Place framework that highlights the importance of place integration and attachment, mobility, social participation and independence. The WHO highlights the importance of age-friendly communities (AFCs) that create inclusive social and physical environments that enable older people to age actively, enjoy good health and continue to participate fully in society [12]. AFCs consider eight domains to support healthy ageing: outdoor spaces and buildings, transportation, housing, social participation, community support and health services, respect and social inclusion, civic participation and employment, and communication and information [12]. Communities and neighbourhoods become increasingly important as people age [13]. Mobility typically centres around the local neighbourhood as distances travelled from the home decrease with age [14, 15], and place attachment increases [16]. Communities with thoughtful urban planning, easily navigable public spaces, and accommodating housing designs can have a significant impact on older adults’ physical and social wellbeing [17]. Socioeconomic deprivation, and consequently, wellbeing, are closely linked to the availability of local facilities and amenities, as limited access to such resources is associated with poorer physical and mental health [9, 18, 19].

Many governments globally, including the Scottish Government, have proposed national planning frameworks that encourage the development of urban spaces to support local living and 20-minute neighbourhoods (i.e. a range of amenities required for daily living within walkable distances from home) [20]. However, the Local Living Framework used in Scotland only makes three explicit references to older adults, suggesting that the needs and perspectives of this demographic may have been underrepresented [20]. Few studies have explored the age-friendliness and suitability of communities for ageing in place. Bosch-Farré et al. [21] explored the perceived barriers and enablers to ageing in place, highlighting the importance of autonomy, well-being, and older adults’ perceived role in their community. Meanwhile, van Hoof et al. [22] explored hindrances in age-friendliness and ageism in design, identifying visible elements of age-friendliness in communication and information, housing, transportation, community support and outdoor spaces and buildings. A study by Tan et al. [23] explored older adults’ perceptions of age-friendliness using the AFC framework through photovoice and semi-structured interviews, highlighting the importance of safety, well-connected essential services and transport to support activity. Similarly, few studies have explored how older adults conceptualise their local area. A study by Milton et al. [24] highlights how older adults perceived neighbourhoods as evolving seasonally and throughout their lives, and associated with social influences from family, friends and community activities, instead of places.

Ageing in Place, and AFCs present two overlapping frameworks to examine how the built environment can support older adults to actively engage with their communities [12, 25]. A recent review highlighted the need for more research on older adults’ accessibility that engages with critical gerontology, and age friendly cities frameworks that incorporates older adults’ voices and experiences [26]. This study aimed to explore mid-to-older-aged adults’ perceptions of their communities and factors influencing age-friendliness, guided by two research questions:

i) What factors shape this population’s perceptions of community age-friendliness of their local areas, and how do these perceptions affect their ability to age in place?
ii) How do perceptions of localness help us (further) understand what matters for ageing in place?

## Methods

### Study design

This study was part of a larger programme of research that explored physical activity and social connectedness in mid-to-older adults (Physical Activity, social ConnectednESs and healthy ageing (PACES) www.gla.ac.uk/paces). The current study reports the second timepoint (6–12 months from baseline) of a longitudinal study, exploring older adults’ experience of physical activity and social connectedness [27]. The first timepoint focused on participant’s social contacts and their physical activity, exploring topics such as their emotional closeness, social support, places visited, current physical activity and life transitions prior to engaging in the study [27]. This second phase was conducted between September 2023 and March 2024. Semi-structured interviews were carried out with adults aged 55–75 years living in the community, and detailed place-based data were collected. Two distinct analyses emerged from this timepoint: the present analysis that explores the age-friendliness of participant’s local areas; and an additional analysis that focused on place accessibility and utilisation, which will be reported elsewhere [28]. Ethical approval was provided by the University of Glasgow College of Medical, Veterinary and Life Sciences College Ethics Committee (project number: 2002184). The reporting of data followed the Consolidated Criteria for Reporting Qualitative Studies (COREQ) guidelines [29].

### Setting

Data were collected from two Scottish local authorities, Renfrewshire and South Lanarkshire, which house approximately 517,000 people out of Scotland’s total population of 5.5 million [30]. Given the aforementioned deprivation-associated inequalities for healthy ageing in place, these regions were chosen based on their high levels of socioeconomic deprivation, high concentration of adults aged 50 years and older, and high population of adults aged over 65 years receiving pension credit, compared to other Scottish local authorities [31]. Additionally, South Lanarkshire is also the first local authority in Scotland to express its intention of becoming an age-friendly community [32].

### Recruitment

Participants for a longitudinal qualitative study (consisting of three timepoints) were recruited from a social network sub-study of PACES (where data were collected on participants, their social contacts, and the places they visit in the community). Participants were originally recruited through methods such as distributing flyers to potential participants across the study areas, with oversampling from postcodes in the lowest income decile. Community partners also shared the study information digitally with individuals in their networks, and advertisements were placed within partner organisations, community centres, libraries and at bus stops. In addition, participants were recruited through advertisements posted on social media [33]. Consenting individuals who fulfilled the sampling requirements were notified about the study and invited to take part by email, telephone or post. Participants were given the option of in-person or remote interviews using Zoom, an internet video-conferencing programme. Participants received a participant information document and privacy notice. Written consent was obtained during the first interview, and audio-recorded consent was obtained prior to follow-up interviews.

### Sampling

Purposive sampling was used to select 25–30 participants from the 140 participants recruited from the larger social network survey, based on demographic data measuring four key characteristics: location (urban or rural), gender, age range (55–64 and 65–70 years), and area-level socioeconomic status (SES), using the Scottish Index of Multiple Deprivation (SIMD) [33]. The present study includes 21 participants who completed the second interview.

### Data collection

During their social network interview conducted at the first timepoint, participants completed standardised instruments to assess physical activity (International Physical Activity Questionnaire (IPAQ), loneliness (Community Life Survey), and frailty (adapted Fried Criteria) [34–36]. Direct measures of loneliness have been shown to capture loneliness better in older adults than indirect measures of loneliness [37]. Qualitative interviews were partially guided by these questionnaires and interactive maps of their local areas populated from digitised Ordnance Survey data (GB National mapping agency), including features such as depicting green space and transport links (Appendix 1). Semi-structured interview guides were created before each interview based on the participants’ social networks and relationships, how participants interact with their local community, their relationship to place, and place-based questions in relation to their social networks e.g., who do you go to this place with?), using the maps to prompt discussion. Place-related questions were informed by the WHO AFC framework, and the Place Standard, a framework used to structure conversations around physical and social elements of places [12, 38]. Interviews were also partially guided by social network maps produced during the social network interviews at timepoint 1. For example, information on the number of social contacts each person listed, attributes of those in the network, and whether they were physically active with each social contact was used to construct person-specific interview questions to follow up on participant-reported social networks and relationships. An interview template is provided in the appendices (Appendix 2). Field notes were completed after each interview, and all interviews were audio-recorded and transcribed verbatim.

### Data analysis

Data were analysed using Framework Analysis, an established systematic approach commonly used in applied qualitative research [39, 40]. It enabled deductive organisation of data according to the AFC and Place Standard frameworks, while also allowing new patterns to emerge inductively across participants and contexts [39, 41, 42]. Transcripts were anonymised and uploaded to NVivo 12 software for analysis. After a period of data familiarisation, a coding framework was constructed based on preliminary coding and categorisation. Two researchers (RT, VJP) independently coded a subset of the transcripts to refine the framework, which was subsequently applied to the full dataset. To assess the consistency of the coding process, approximately 20% of transcripts were double-coded (RT and VJP). The analytical process followed a layered approach combining both deductive and inductive elements:

- Layer 1: Mapping age-friendliness
- In the first layer, data were deductively coded against the AFC and Place Standard frameworks [12, 38], identifying key barriers and facilitators to age-friendliness within each domain. AFC domains grouped into three categories [43]:
  i. Physical Environment: Outdoor Spaces and Buildings, Transportation, Housing.
  ii. Social Environment: Social Participation, Respect & Social Inclusion, Civic Participation & Employment.
  iii. Service Environment: Communications & Information, Community Support & Health Services.
- Layer 2: Exploring ageing in place
  The second analytical layer examined how these barriers and facilitators related to participants’ experiences of ageing in place, guided by Bigonnesse and Chaudhury’s [25] multi-faceted framework. This framework provided a person-centred and capability-based lens encompassing Place Integration and Attachment, Mobility, Social Participation, and Independence, allowing us to contextualise age-friendliness within lived experiences of place.
- Layer 3: Emergent concept – perceptions of localness
- The final layer adopted an inductive approach to explore emergent meanings of localness as described by participants. This layer captured participants’ perspectives on how local environments, relationships, and identities shaped their experiences of community and place.
- This layered approach allowed the analysis to connect structural and experiential dimensions of ageing, linking the tangible features of age-friendly environments with the ways people experience and make sense of place. Henceforth, age-friendliness will refer to place-based age-friendly characteristics.

### Research team and reflexivity

The lead author is a male postdoctoral researcher experienced in qualitative research, with a clinical background in Physiotherapy, who led the analysis but did not conduct the interviews. This distance from the data collection process allowed for a degree of detachment from the analytical process during coding and interpretation. This also required clear communication with the three members of the research team (BR, MT, VJP) who delivered the semi-structured interviews as well as reviewing their field notes, to ensure that contextual nuances were understood and incorporated into the analysis. The author group was gender balanced and included junior and senior researchers from a variety of disciplines, with extensive experience with older adults, health psychology and behaviour change. This multidisciplinary composition enriched the study by bringing diverse theoretical and methodological perspectives to bear on data interpretation, particularly regarding constructs like social connectedness and motivation for physical activity. The team represented various life stages, from early adulthood to late middle age. Members of the research team did not have any pre-existing relationships with the study participants. Participants were told that these interviews would explore physical activity and social connectedness over time, with the second timepoint focusing specifically on their local environments.

### Public involvement

A Community Advisory Group, comprising 15 mid-to-older aged adults, including individuals from the study areas, local community organisations, and national older adult associations, provided input and feedback on the interview guides, analysis and interpretation of findings. For example, the issues surrounding public transport were highlighted by the advisory group, particularly in rural areas.

## Results

### Participant characteristics

Participant demographics are summarised in Table 1. Twenty-one (15 females, six males; mean age 65.0 ± 5.5 years) of the 27 participants recruited during the first timepoint completed the second interview. The six individuals who did not participate in the second interview were demographically similar to those retained, although more males withdrew (5 M:1 F). The sample reflected a mix of urban and rural settings, varying levels of socioeconomic deprivation, and a range of physical activity, loneliness, frailty and educational backgrounds.

**Table 1:** Demographic characteristics of the recruited sample

Characteristic		Total (*n* = 21)	
Sex	Female	15	71.4%
	Male	6	28.6%
Age (Years)	55–59	4	19.0%
	60–65	6	28.6%
	> 65	11	52.4%
Ethnicity	White	21	100%
Highest Educational Achievement	Completed Secondary School	9	42.9%
	Completed College or University	5	23.8%
	Completed Postgraduate Qualification	6	28.6%
	Not reported	1	4.8%
Area	Renfrewshire	7	33.3%
	South Lanarkshire	14	66.7%
Urbanicity	Rural	4	19.0%
	Urban	17	81.0%
Area-Level Deprivation Quintile (SIMD)	1 (most deprived)	6	28.6%
	2	4	19.0%
	3	4	19.0%
	4	4	19.0%
	5 (least deprived)	3	14.3%
Physical Activity (IPAQ)	Low	5	23.8%
	Moderate	9	42.9%
	High	7	33.3%
Loneliness (UCLA Loneliness Scale)	Never	9	42.9%
	Hardly Ever	4	19.0%
	Sometimes/Occasionally	7	33.3%
	Often	1	4.8%
Frailty (*n* = 20) (Fried Frailty Criteria)	1 (pre-frail)	1	5.0%
	2 (pre-frail)	12	60.0%
	3 (frail)	5	25.0%
	4 (frail)	1	5.0%

### Layer 1: age-friendliness of communities (framework-informed analysis)

This first analytical layer draws upon participants’ accounts of the age-friendliness of their local areas, using the AFC and Place Standard frameworks to guide initial coding. Summaries of the identified barriers and facilitators to age-friendliness with supporting quotes are provided in Table 2. Identified barriers applied to all AFC framework domains, highlighting the perceived widespread deterioration of these communities.

**Table 2:** Identified supports and barriers to the age-friendliness of communities charted to the age-friendly cities, and place standard domains

Age-Friendly Cities Domains		Place Standard Domains	Identified Barriers and Supports	Illustrative Quotes
Physical Environment	Outdoor Spaces and Buildings	Streets and Spaces Natural Space Play and Recreation Care and Maintenance	Barriers: Poorly maintained paths, antisocial behaviour, poor accessibility and overcrowding hinder outdoor space usage. Barriers such as parking on pavements, or dangerous drivers further limit accessibility. Poor maintenance of public spaces discourages use.	*“There was an awful lot more green spaces… I mean*,* there is for instance*,* what was called the (town centre) park. It’s just a dump now. It’s an absolute dump.”* P7-61 M-SL
			Facilitators: Outdoor spaces should be accessible, safe, and engaging, with well-maintained paths, scenic natural areas, and multipurpose recreational zones that are affordable and inviting for various activities. Regular care, such as paving, de-icing, and tree trimming helps ensure these areas remain accessible year-round.	*(Referring to park) There’s probably bits that they wouldn’t do because they’re really hilly and have steps and stairs. Strathclyde Park is really flat; so*,* you get people out with prams and kids out with wee bikes. It’s really accessible.”* – P17-66 F-SL
	Transportation	Moving Around Public Transport Traffic and Parking	Barriers: Poorly maintained paths, challenging terrain, limited resting opportunities, and a lack of facilities like public restrooms, can make moving around the local area difficult. Public transport is hindered by high costs, congestion, unreliable services, limited routes, and accessibility concerns, further reducing convenience for older adults. Traffic congestion and limited available parking limits car use.	*“Without my car*,* I’m housebound. Because even for me to get to a bus stop*,* without the hazards of me trying to get on and off a bus with a driver that doesn’t let you sit down before he pulls away*,* is on the main road…. I can’t walk that far. So*,* I can’t get a bus.”* P1-57 F-R
			Facilitators: Accessible, well-maintained paths and reliable public transport, with nearby stops and affordable fares can enhance mobility and social engagement. Additionally, dedicated bike lanes, free parking, and minimal traffic congestion support active lifestyles and provide convenient access for diverse transportation needs. Participants felt safe walking home at night, walking in parks, or near train/bus stations.	*“My pals and I*,* we just jump on and off them [buses] and…you know*,* you’ve got your wee bus pass and you can scuttle about and…aye*,* it’s great.”* P12-61 F-SL
	Housing	Housing and Community	Barriers: No opportunities to downsize homes. Houses not suitable for older adults - require adaptations for mobility aids, with stairs challenging. New homes being built in local area replacing green spaces and increasing strain on existing services. No access to facilities or services near home. No sense of community post-COVID, bereavement of neighbours and replaced by new families. Antisocial behaviour by neighbours.	*“In ten years’ time*,* I’ll be*,* what*,* 76. Hopefully a fairly fit 76-year-old*,* but that might not be the case*,* so I’d like to lose some of the stairs and that’s why we’ve been investigating Ayrshire.”* P4-65 F-SL
			Facilitators: Sense of community in their local area. They know the area and people well. Neighbours are respectful. Community supported older adults during COVID (delivered groceries, called round to homes).	*“The people look out to make sure you’re okay and you’re safe and you’re sound. That’s about it. It’s quite nice. People are very nice. Neighbours are nice. Feel quite comfortable here.”* P16-63 F-SL
Social Environment	Social Participation	Social Interaction	Barriers: Declining access to sports facilities, social venues, healthcare, with shops reducing hours or closing. Must travel further to access essential services and facilities. Newly built homes have led to increased demand on existing services.	*“There is nothing…. Nowhere you can really go other than the park and*,* you know. You can’t go to the park when it’s pishing down with rain.”* P12-61 F-Ren
			Facilitators: It is important to have affordable, accessible, enticing facilities, available including sports clubs, social venues, educational institutions, healthcare and shopping options. Multi-purpose centres such as churches and community centres can encourage physical and social activities, with intergenerational connections promoting community cohesion.	*“I mean*,* there are the places there*,* I guess*,* if you go and find them*,* you know*,* to meet people. So*,* you know*,* there are sport centres*,* and there’s leisure centres*,* and there’s the church centres*,* so there are places that you can go.”* P10-55 F-SL
	Civic Participation & Employment	Work and Local Economy	Barriers: Certain jobs promote sedentary behaviour, and can be time consuming, leaving less freedom to do other activities. Services propped up on voluntary labour, concern that they may cease without volunteers.	*“The residents’ association closed down ‘cause nobody would take it on.”* P12-61F-Ren
			Facilitators: Employment and volunteering can facilitate increased physical activity and social interaction. Volunteering can be rewarding or fulfilling and can help mitigate the loss of role following retirement by providing structure and organisation to people’s day. Promotes intergenerational connections.	*“So*, *health charity* *tries to do that for people (connect people)*,* because it’s not happening the way it used to happen… I’m there to support them*,* and*,* you know*,* they make their own friends and connections*,* and they don’t need you anymore.”* P5-68 F-SL
	Respect and Social Inclusion	Identity and Belonging	Barriers: Some local areas described as not having sense of community (particularly post covid). People keep to themselves, no community cohesion present. Activities are aimed at age, instead of level of ability, which may perpetuate ageist sentiments whilst also discouraging intergenerational connections, and involvement from middle-aged adults.	*“It’s maybe not like it was back years ago*,* when people would be…I think know their neighbours better. I mean*,* my mum could probably have named everyone in the street.”* P8-67 F – SL
			Facilitators: Involvement in community groups or resident groups provide platform for older adults to raise issues relevant to them. Available activities help promote physical activity, social interaction, and intergenerational connections. Discounts available for older adults to encourage involvement and participation.	*“Saturday morning is a bootcamp*,* and there’s a big crowd of girls*,* and it’s great fun. And I’m the oldest by 30 years… I’ve gone to various gyms before*,* and you can just go and sit on the machine and go and it’s boring. With this crowd of girls*,* it’s not competition.”* P21-73F-SL
Service Environment	Communication and Information	Information Language Computer and Internet	Barriers: In-person methods of providing information (e.g. flyers, posters etc.) may not reach older adults. Digital exclusion can prevent older adults from accessing information online, reducing awareness of activities happening or available in their local area. Additionally, ageist sentiments can discourage engagement, as does inconvenient booking processes.	*“When I first moved to Paisley*,* the Paisley Centre*,* half dead Paisley Centre*,* had lots of notices about what was happening*,* and you could take flyers and stuff. I don’t see those. They don’t seem to be there anymore.”* *P20-64 F-Ren* “*They looked at us*,* there’s people there from*,* well maybe*,* what*,* 60 to 75… they go*,* oh*,* better be careful*,* you know*,* in case somebody dies on me.”* P17-66 F-SL
			Facilitators: Information readily available both in-person (via notice boards, flyers etc.), from healthcare professionals, through word of mouth, and via social media and community groups. Activities are well advertised and are inclusive, and easy to book.	*“We tend to get a lot of post-its and flyers through the door*,* which*,* you know*,* isn’t quite the high-tech of the internet*,* but sometimes low-tech is just as good.”* *P11-61 M-Ren*
	Community and Health Services	Facilities and Services	Barriers: Difficulties accessing healthcare services due to long wait times, inability to get appointments, needing to travel for specialist care, or needing to go privately. Overworked healthcare staff and overwhelmed healthcare services can discourage health seeking behaviour. Essential services such as banks closing physical branches and transitioning to online services reducing accessibility for older adults.	*“I have a health centre literally on my doorstep*,* but you cannot get an appointment for love nor money”* P10-571-SL
			Facilitators: Healthcare services are accessible and have short wait times making it easy to get appointments. GPs referring to Allied Healthcare Professionals such as physiotherapists and pharmacists to decrease wait times. Positive past experiences encourage older adults to seek help. Involvement in community organisations provides opportunities to share issues they may be facing.	*“My social network is of similar age to me and older. And so they’re using the NHS services quite a lot and they’re getting appointments quickly. They’re getting seen*,* triaged… I know they’re stretched*,* but they are remarkably doing what they’re required to do.”* P4-65 F-SL

#### Physical environment

Participants from both Renfrewshire and South Lanarkshire described a gradual decline of their local environments over time and as they aged, which impacted their mobility, independence and their physical and social opportunities. Reported barriers for transportation and mobility included limited parking, poorly maintained paths, blocked pavements, and unsafe crossings, which particularly affected those with mobility impairments. Some described how housing options in their local area were largely not age-friendly, with stairs being described as problematic and having limited opportunities to downsize or adapt their homes. In some areas, green spaces were reportedly being replaced with new housing developments aimed at families, which led to increased strain on existing facilities and reduced opportunities for physical and social activities. Access to parks, well-paved paths and reliable public transport facilitated healthy ageing.

#### Social environment

Social participation and inclusion were seen as vital to wellbeing but had diminished in the post-COVID landscape, due to declining access to shared spaces and reduced community cohesion. Participants reported fewer opportunities for intergenerational connection, with activities often narrowly targeting older adults. Social opportunities were reportedly further limited by affordable venues reducing their hours, increasing their prices or closing entirely. Reliance on voluntary labour raised concerns regarding the sustainability of community groups. Where community groups remained active, participants reported increased social engagement, highlighting the impact of accessible and welcoming spaces.

#### Service environment

Participants’ capacity to stay active and independent was influenced by the provision of services and information in their local areas. Barriers included complex booking systems, digital exclusion, long waitlists and limited in-person options, which reduced access to essential services such as healthcare services and banks. Closure of local facilities increased travel distance to access similar resources. Clear information and in-person provision helped older adults feel informed and supported.

### Layer 2: ageing in place (integrating experiences across domains)

While the AFC framework helped identify specific barriers and facilitators, participants’ accounts reflected broader experiences of ageing in place. In this second layer of analysis, the previously identified barriers and supports are examined in relation to four interconnected domains of Ageing in Place: Place Integration and Attachment, Mobility, Independence, and Social Participation, as outlined by Bigonnesse and Chadhury [25].

### Place integration and attachment

The Ageing in Place framework highlights the importance of the home and neighbourhood environments in later life [25]. Place attachment develops through long-term residency, fostering strong emotional ties to a local area, whereas place integration is the “active process that connects person and place, and continually transforms them and their relationship” with said place [44]. Participants reported being attached to their communities, of which many were longstanding members: “*I’ve lived here my whole life. Growing up in (Name of Town) as a new town was absolutely wonderful”* (P7-61 M-SL). However, several participants expressed that their homes and neighbourhoods were unsuitable for ageing healthily in place, which has weakened their sense of place attachment, with some considering relocating or downsizing. Physical factors, such as accessibility issues and stairs becoming more challenging with age, and social factors, such as children moving out, were common reasons: “*We’re currently living in a house that used to house five adults at one point. Now*,* it’s just two of us. We probably don’t want to carry on living in for the next ten to 15 years*,* because it’s got a lot of stairs in it.”* (P4-65 F-SL). However, participants reported that there are few age-appropriate options for them to relocate to: *“There is no real opportunity for people to downsize when we get older and go into*,* like*,* a flat.” (*P9-70 M-Ren). Although new homes are being built, these homes were primarily geared towards families, and replaced green spaces in participants’ local areas which has placed increased strain on existing resources: “*The green belt has gone and they’re building new houses left*,* right and centre…They’ve built one primary school*,* but no shops*,* and I’m not sure (name of area) is really capable of taking the amount of traffic there might now be…”* (P4-65 F-SL).

### Mobility (travel and transport)

Mobility, as outlined in the Ageing in Place framework, involves physical movement, including walking (with or without mobility aids), driving, or using public transportation, to meet daily needs and participate in activities [25]. Key considerations included accessibility, public transport, and access to resting opportunities and public bathrooms (Table 2). Participants described declining public transport services, which were unreliable, infrequent, slow, meandering and costly: *“I tend to very rarely get the bus because quite frankly I could probably walk to Glasgow quicker… I could cycle in half the time”* (P7-61 M-SL). While some initiatives, like free bus passes for adults aged ≥ 60 years were well-received – “*It doesn’t cost you anything. I love my bus pass. That was the best thing about being 60”* (P12-61 F-Ren) ***–*** others, such as a new cycle path project, faced criticism: *“They didn’t use the existing network that was there… and created cycle paths everywhere*,* that actually are not particularly cycle friendly”* (P10-55 F-SL). Safety concerns around bus and train stations, especially anti-social behaviour at night, discouraged public transport use: “*The train station used to get… a lot of trouble down there. And if I was out and coming back later my husband used to pick me up” (P17-66 F-SL)*. Declining mobility also affected place attachment, as it highlighted the need for ample storage for mobility aids: *“The next option is then the wheelchair… and I haven’t got the storage in my house to store the wheelchair”* (P1-57 F-Ren). Further perspectives are provided in the subsequent layer.

### Independence

The Ageing in Place framework defines independence “as the capacity to exert control on one’s environment, to make decisions and choices, and to meet daily needs”, and emphasises the role of meaningful social connections, social support from friends and family, and social participation in organisations or local governments [25]. Independence is impacted by older adults’ mobility, social support and participation, the proximity of services and the accessibility of the built environment [25]. The closure of essential services, and shifts to online access, such as banking, have reduced older adults’ independence for those not technologically skilled as it forces a reliance on others: *“I’m not that tech savvy so [Friend’s] going to help me with it. She set it up…or I set it up and then she jiggled it all about…there’s a lot of old people here that don’t (understand technology)”* (P12-61 F-R).

Physical decline can further increase reliance on others, particularly for those with mobility impairments, in order to move around their local areas: *“Once we’d had our meal*,* we then wanted to go somewhere else which would require a bit more walking for me. His answer was*,* well*,* you just phone me*,* I’ll come and get you and I will lift and lay you to the next place… But that’s a heavy reliance on other people” (P1-57 F-Ren)*. Community cohesion during the COVID-19 pandemic provided vital social support, through initiatives such as home deliveries of groceries, helping maintain independent living: *“During COVID the wee local shop they were taking telephone orders*,* and they were doing deliveries which they don’t normally do… So*,* that was quite good.” (P9-70 M-Ren)*. Social support from loved ones and communities can help older adults age in place, but reliance on others can pose key challenges when that support is not available: *“Public transport is a no*,* I just can’t do it*,* unless I am with someone who I have a lot of confidence in.” (P1-57 F-Ren).*

#### Social participation

Social participation enables older adults to engage in community life through volunteering, work, civic involvement, and intergenerational activities [45, 46]. Participants valued volunteering as it was rewarding and helped mitigate the loss of role following retirement: “*I’ve been a volunteer since then [retirement]*,* because I wanted to do something constructive*,* so I’m involved in a local health charity”* (P5-68 F-SL). Similarly, involvement in community groups provided platforms to raise issues concerning them: *“I’m also a public partner with (Name of National Organisation)… I don’t want to just do things locally; I like to do things further afield”* (P5-68 F-SL). Additionally, employment and volunteering opportunities were helpful in promoting intergenerational connections: “*My older sister and I both do volunteering for a local health charity*,* she was involved with older people…whereas I’m involved with younger families”* (P5-68 F-SL). However, there was a sentiment that some services or groups were being propped up on voluntary labour from older adults, and without such labour, the activities would cease: “*If you’re involved in a Sporting* *club*,* if you end up being a coach*,* the secretary and you’re doing three jobs*,* you end up breaking*,* and if you’re doing three jobs and you get burnout*,* the club collapses”* (P4-65 F-SL). The opportunity cost of such roles must also be considered, as involvement can be time-consuming, leaving less freedom to do other activities: *“I would say*,* that the club*,* the role of the club secretary is impinging on my freedom*,* if you want to put it that way”* (P4-65 F-SL).

### Layer 3: emergent concept – perceptions of localness 

This final layer explores how participants perceive and experience “localness”, and how this shaped their attachment to place. We describe how the frameworks of age-friendliness and ageing in place (outlined above) are reflected, or challenged, in everyday life. Participants described localness in terms of how easily they could move around, access essential services, and remain socially connected.

####  Mobility (moving around)

Being able to move around was central to feeling connected to their local area. Mobility shaped not only access to services but also a sense of independence and belonging, key aspects of ageing in place and of age-friendly environments. For some, localness was defined by their preferred or available modes of transport. Drivers and cyclists described broad local areas:

*“The whole of (Name of Town)*,* is accessible*,* because I’ve got a car.”* |P10-55 F-SL.

*“It’s easy to get to any of the places I want to get to within (Name of Town)*,* because I’m fit*,* and I can cycle.”* |P4-65 F-SL.

Conversely, those who didn’t have a car or predominantly walked described a narrower perspective on localness, framed in terms of distance or duration:

*“Local is*,* kind of*,* walking distance. If we take local as being*,* say*,* a two-mile radius… it’s just about a 15 minute walk the other way.”* |P7-61 M-SL.

However, this perception of localness hinges upon an individual’s ability to mobilise safely and effectively within their community, which participants described as being influenced by structural factors such as accessibility of the built environment:

*“Was walking… going round to the station*,* actually going to meet friends in Glasgow*,* and it was an uneven surface*,* and I went right down.”* |P5-68 F-SL.

Or by individual factors such as declining physical health and well-being:

*“I still cannot walk as far as I could*,* partly because of my ankle but partly because of the pain that comes in my back from it not being supported obviously when I’m walking.”* |P1-57 F-R.

As their health declined, some described a shrinking of their local areas, where places once easily reached became inaccessible:

*“We could walk a fair distance*,* you know*,* and we’d go away somewhere and walk*,* but…so*,* it’s been a bit*,* you know*,* of a downer*,* really*,* just*,* kind of*,* I can’t do that*,* so yeah.”* | P8-67 F-SL.

This reduced capacity to walk can force reliance on other modes of transport, notably driving:

*“That would have been walking distance for me before*,* but now not*,* I would always have to go in the car.*.. *I have to drive everywhere really; there isn’t anywhere that is within walking distance.”*|P1-57F-R.

However, reduced physical mobility and the resulting smaller local area size can limit access to other forms of transport, especially when they require the ability to walk a certain distance, making public transport impractical:

*“It’s the walking aspect*,* he couldn’t do it*,* and you can’t really load the scooter on. So*,* that would stop us getting public transport.” (referring to partner)*|P17-66 F-SL.

Even when public transport is available, small actions, like drivers not waiting for someone to sit down before moving off, can make public transport feel unsafe, reducing confidence and discouraging use:

*“I can’t do buses*,* getting on and off buses*,* on crutches is an absolute nightmare… they don’t let you sit down before they pull away and then you get that jolt.”* | P1-57 F-R.

This reduced mobility and decreased accessibility of their surroundings leads to a shrinking of one’s local area and fear avoidance, and can result in reduced social engagement:

*“She fell on Saturday when we were out. It’s*,* kind of*,* knocked her confidence.” (referring to friend)* |P12-61 F-Ren.

These accounts illustrate how mobility influences both physical and mental wellbeing. When participants felt secure and supported, their sense of localness expanded. When mobility declined, localness contracted, sometimes to the point of isolation.

#### Essential services

Accessible and affordable community and health services, such as shops, pharmacies, and community facilities, play a crucial role in keeping older adults healthy, independent, and active. For some participants, localness was defined by how far they had to travel to reach these essential services. While some described a small, easily walkable area:

*“There’s four high flats and six maisonettes. We’ve got… two wee rows of shops. We’ve got everything that we need here. Everything that we need.”* |P12-61 F-R.

Others had a broader sense of localness shaped by their ability or need to travel further:

*“It depends what you mean by local. I mean*,* I consider Glasgow as local* (40 miles from his town).” |P7-61 M-SL.

Localness was shaped not only by geography but by familiarity, as places visited often came to feel local even when distant, with access to such services framing localness and underpinning daily movement:

*“There’s a couple of supermarkets*,* an Aldi in (Name of Local Area)*,* so I go up there. Also*,* the doctor’s surgery…and I have to get the prescription every week from the local chemist. So that’s probably how I see local that way.”* |P19-57 M-R.

Many participants reflected on the effects of service decline, particularly the closure of shops in town centres. Reduced availability of amenities often meant travelling further, which could be difficult for those with limited mobility or who didn’t drive:

*“There’s also the main shopping centre. You know*,* which has got… I was going to say a variety of shops*,* not now it doesn’t.… like everywhere now*,* there’s really not as many shops.”* |P14-66 F-SL.

Factors such as closures, parking fees and price increases have forced people to travel further away to access essential services:

*“People will go to (Shopping Centre) because it’s free parking. You know*,* rather than go into (Name of Town) to pay for parking. They shot themselves in the foot with that one.”* |P7-61 M-SL.

Again, ambulation becomes a focus point, where access to local amenities is reliant upon the ability to mobilise safely and effectively:

*“I love the Co-op [shop]*,* over in the village*,* because I can walk there*,* it’s handy*,* it’s kind of*,* you can get everything you need.”* |P10-55 F-SL.

However, practical challenges, like carrying heavy bags or navigating uphill terrain, can make even short trips difficult:

*“And I wouldn’t carry*,* like*,* a heavy bag up that road because it is uphill.”* |P17-66 F-SL.

These access constraints had social as well as practical consequences. Participants who could still walk to local shops described regular informal contact with neighbours and friends:

*I always meet someone when I’m out*,* you know*,* even at the supermarket*,* you know*,* you stand and chat. And my husband will say*,* where have you been*,* you know*,* talking to someone in the supermarket for an hour”* |P8-67 F-SL.

Participants who depended on driving, or who no longer made regular trips to local shops, described experiencing fewer spontaneous social interactions:

*“I’m not sure that’s good for people*,* for communities*,* because people want to go to their wee*,* local shop*,* you know….”* |P10-55 F-SL.

Meanwhile, the closure or inconsistency of community facilities affected participation in leisure activities, with some willing to travel further to sustain valued routines:

*“I wouldn’t think twice about travelling to it.”* |P4-65 F-SL.

Whereas others disengaged entirely when facilities repeatedly opened and closed:

*“I used to (go to places)* *but they keep opening and shutting and things… we don’t go very often now.”* *P9-61 F-R.*

In addition to supporting basic needs, access to essential services can help maintain social connections, daily routines, and engagement with the community. Reduced access can limit independence and opportunities to stay connected and involved locally.

#### Social connection

Participating in leisure, social, cultural, and spiritual activities can help older adults stay engaged and connected. For some, localness was defined less by proximity and more through these social ties, such as where they knew people, socialised, or regularly visited loved ones. As one participant described:

“*But locally*,* I mean*,* if it was knowing neighbours and things like that*,* I mean*,* there probably is quite a circle of folk that I know quite well in that*,* just in that small section*,* just in the street.”* |P8-67 F-SL.

Social identity also shaped how people oriented themselves locally, with some participants describing familiar social spaces they returned to because they felt known and comfortable there:

*“I tend to do most of my socialising*,* or more my area I stay is the east end. This area here. I don’t tend to venture much to the west end.”* |P11-61 M-R.

For others, localness expanded outward to areas where family lived, reinforcing that place attachment can be influenced by relationships as well as physical proximity:

*“I would say Hamilton’s local. The whole lot… I’ve got one son stays in (Town 1)*,* one daughter stays up (Town 2). Other one stays round there. And my oldest boy stays in (Town 3).”* |P18-74 M-SL.

These accounts illustrate that localness is flexible, shaped by social ties, routines, mobility, and belonging. However, changes, such as closures of community spaces, declining mobility, or limited public transport, can disrupt these connections:

*“The deficiencies in some older people living in the outskirts of town where public transport isn’t so good*,* you’re actually cutting off one of their lines of social interaction.”* |P4-65 F-SL.

Another reflected on how generational turnover is changing their perception of the social composition of their communities:

*“I’ve been here for 30 years. Most people here are in my age bracket*,* but there are young families moving in; when people*,* kind of*,* die off”* |P17-66 F-SL.

Additionally, some felt that the COVID-19 pandemic had lasting effects on social cohesion:

*“I think a lot of these areas have lost that sense of community.”* |P1-57 F-R.

Together, these reflections highlight how localness is not a fixed concept, but instead one that is continually changing according to health status, social networks, infrastructure and broader societal change.

## Discussion

This study expands existing knowledge of ageing in place through the concept of ‘perceived localness’ by highlighting how mobility, service provision and social ties influence place attachment, providing novel insights into how mid-to-older adults perceive their local areas. By exploring participants’ perceptions, we address how older adults conceptualise localness and the barriers and facilitators to ageing healthily within these areas. Deterioration in the physical, social and service environment (as charted to the AFC and Place Standard frameworks) was reported in both local authority areas in Scotland and presented significant challenges to ageing healthily in place. Barriers were often interconnected, affecting multiple domains simultaneously and should not be considered in isolation. Participants experienced declining place attachment, mobility, independence, and social participation, limiting their abilities to remain active and engaged members of their communities. The Scottish Government’s National Planning Framework for Local Living and 20-minute Neighbourhood guidance highlight both the quality of the services, amenities and facilities and the community experience in accessing them, in addition to their quantity and proximity to home as key characteristics in creating places where people can live locally for a range of health and social benefits [20]. However, participants identified several barriers to local living, and consequently, ageing healthily in place, according to their perceptions of ‘localness’. The implications of the identified barriers to the provided perceptions of localness will be discussed below.

Access to essential services and mobility were key concepts described by participants to conceptualise localness. Participants described localness not only in terms of geographic proximity, but also ease of access, perceived safety, and familiarity with their environment, highlighting that localness is shaped by both individual capabilities and community infrastructure. This study highlights a simultaneous shrinking and widening of local areas occurring with increasing age, as travel distances decreased with declining health, and the closure of essential services forcing longer travel (for those with access to cars). Certain barriers to safely mobilising reported by participants, such as poorly maintained pavements, hilly terrains, declining physical health and fear of falling, have been echoed previously [47–50]. Such barriers heavily affect older adults with mobility impairments, and inaccessible transport options can make 20-minute neighbourhoods unsuitable for an ageing population [20]. These policies often consider proximity and not the more detailed nuances of the built environment, like poor-quality streetscapes, that can facilitate or hinder older people’s ability to walk to local destinations and access public transport. Urban design policies must consider the contextual aspects of the built environment that can support or limit older people from benefiting from these national planning policies. The Local Living framework suggests that remote or digital access to services may reduce travel needs and widen access [20]. However, with 18% of people aged ≥ 65 years not using the internet for personal use or having no access to the internet [51], such recommendations may actually further inequalities and limit access in this population [52]. As essential services close, participants reported needing to travel further to access physical branches of services like banks, which may force reliance on public transport, which was viewed as inaccessible by some participants, or informal support like being driven by family, which can impact older adults’ sense of self [15].

Social participation and place attachment were central aspects of the Ageing in Place framework [25], and were important to participants’ perceptions of localness. These social connections shaped participants’ sense of localness, defining where they felt ‘local’ based on relationships, community engagement, and familiarity. Place attachment considers an individual’s relationship with others in settings which intertwine with their lives [53]. This study builds upon findings by Gardner et al. [54] on the importance of family and neighbourhoods, and incorporates Clarke et al. [55]’s demonstration of how social capital, and home and neighbourhood satisfaction influence preferences for ageing in place. However, the parameters by which participants described this social connection, such as where they know people and where they socialise, are rapidly changing. Participants discussed the dwindling sense of community following COVID-19, the generational shift following the bereavement of neighbours, and the closure of social spaces, reducing opportunities for social connection and the benefits of such [56, 57]. Place identity, social cohesion, social relationships and a sense of belonging in the neighbourhood are significant predictors of social and physical wellbeing in older adults [58, 59]. Social isolation and loneliness in this population can result in a rapid decline of motor function, and the development of physical frailty in individuals not previously physically frail [60], limiting their abilities to actively engage in their community. With approximately half of people aged ≥ 60 years being at risk of social isolation and loneliness [61], the changing social landscape for older adults is a pressing issue.

The Place Standard, AFC and Ageing in Place frameworks were useful frameworks for evaluating the built environment for older adults, and their combined use may address the limitations of any one framework alone. Although not specific to older adults, the Place Standard evaluates a broad assessment of physical and social factors affecting a place [38]. In contrast, the AFC framework specifically focuses on the needs of older adults, such as social inclusion, housing, and community support [12]. However, the AFC can be more policy-driven and less grounded in local processes. Meanwhile, the Ageing in Place framework offers a person-centred focus, highlighting the importance of autonomy and continuity in home settings [25], but it may overlook broader environmental and infrastructural factors. Used together, these frameworks provide a layered approach: the Place Standard provides an overall baseline; the AFC adds a targeted age-friendly framework; and Ageing in Place incorporates the lived experiences and preferences of older adults. Combining these frameworks can enable policymakers and communities to create environments that are inclusive, accessible and responsive to the varying needs of ageing populations.

The findings of this study also expand upon the physical, social and service environments of the AFC framework, by highlighting that they are not only external domains of influence, but also central to how participants conceptualised or perceived localness through their experiences of mobilising, accessing services and maintaining social connections. Additionally, this elastic view of localness highlights the importance of continuous improvement and responsiveness in AFCs to remain attentive to changes in the physical, social and service environment, and to how these shifts shape older adults’ lived experiences of their local area. Equally important is including older adults’ perspectives in planning and policy processes to ensure their environments support their needs and preferences throughout older adulthood. Additionally, although intended for use as a survey, by exploring the domains of the Place Standard qualitatively [38], this study sheds light on how participants felt about their local areas. Participants proposed strategies to improve public transport accessibility and local walkability, such as adding more bus stops to reduce walking distances, ensuring buses wait until passengers are seated, well-paved roads to accommodate mobility aids, providing resting stops and ensuring access to public toilets, echoing findings of a previous review [62]. Such strategies may improve the ability to travel within their communities and healthily age in place [15], while promoting improved physical and mental health and social connectedness [63].

### Strengths

The recruited sample varied according to levels of physical activity, loneliness, deprivation, rurality and frailty, providing broad perspectives on healthy ageing in two Scottish communities, addressing limitations described by Bosch-Farré et al. [21]. Additionally, by mapping participant responses to the Place Standard and AFC frameworks, a structured examination of the age-friendliness of the communities is provided. By documenting the barriers and enablers to age-friendliness, pitfalls to healthy ageing and strategies to address them can be identified. Additionally, using Ordnance Survey Points of Interest data allowed participants to guide interviewers through their local areas and structured the discussions.

### Limitations

Although the sample varied according to the aforementioned criterion, the recruited sample consisted solely of white people, and mainly women, identifying as Scottish, likely due to the fact that neither local authority is very ethnically diverse. As such, the perspective of minority ethnic older adults towards the age-friendliness of these communities has not been gathered, and the transferability of findings to that population is limited. A recent report by Ambition for Ageing highlights several barriers to ageing in place for minority ethnic communities, including language barriers, complex health and care needs, limited trust in mainstream services, concerns of crime and service closures [64]. Broader recruitment strategies are recommended to capture diverse perspectives on community age-friendliness.

## Conclusions

Understanding age-friendliness involves recognising the challenges older adults face, supporting their ability to stay engaged in their communities, and promoting their physical and mental well-being. Reported barriers to age-friendliness applied to all domains of the AFC and Place Standard frameworks, highlighting how deficits in the built environment limit older adults’ abilities to healthily age in place. Safe mobility and access to transport become increasingly vital as physical capacity declines and local services close, while a declining social landscape undermines place attachment and well-being. Although policies may have broad societal goals that seek to create AFC’s, often they lack the specificity to deliver these at local levels. Through these interviews with older people, specific and local actions to improve local communities and make services more accessible have been suggested. Policy makers should ensure that place-based approaches engage with local communities to consider how best to plan collaborative place-based action, using frameworks to support these collaborative, multisectoral, place-based approaches [65].

## Supplementary Information

Supplementary Material 1.

Supplementary Material 2.

## Data Availability

The data that support the findings of this study are available from the corresponding author upon reasonable request.

## Abbreviations

AFC: Age-Friendly Cities
COREQ: Consolidated Criteria for Reporting Qualitative Studies
IPAQ: International Physical Activity Questionnaire
PACES: Physical Activity, social ConnectednESs and healthy ageing study
SES: Socio-economic status
SIMD: Scottish Index of Multiple Deprivation
WHO: World Health Organization
