# Supplementary figures and images for: Healthy ageing in place: perspectives on age-friendliness in ‘local’ communities
Source: BMC Public Health. 2025 Dec 23;26:337. doi: 10.1186/s12889-025-26050-4 (PMC12837096; doi:10.1186/s12889-025-26050-4)

**
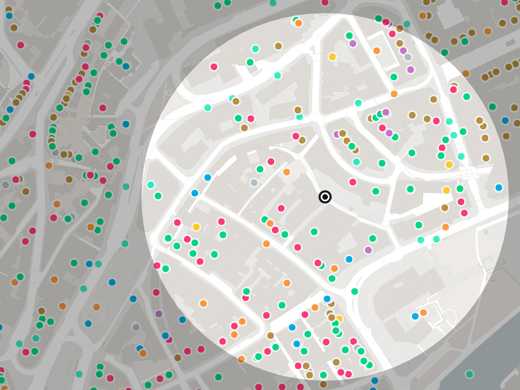
**

Supplement: Supplementary file 1 — Supplementary Material 1. [file 12889_2025_26050_MOESM1_ESM.docx]
