# Supplementary material for: Healthy ageing in place: perspectives on age-friendliness in ‘local’ communities
Source: BMC Public Health. 2025 Dec 23;26:337. doi: 10.1186/s12889-025-26050-4 (PMC12837096; doi:10.1186/s12889-025-26050-4)
Supplement: Supplementary file 2 — Supplementary Material 2. [file 12889_2025_26050_MOESM2_ESM.docx]

**PACES: LQI Interview Guide – Timepoint 2**

**Participant Info**

**Data requirements:** Researcher and participant should have access to baseline SNA figures, and the online ‘place map’.

<https://glasgow-uni.maps.arcgis.com/apps/instant/basic/index.html?appid=d8d4b963704b40939ca86f88ec6bce1c&locale=en-GB>

**Prior to the interview**

[Researcher to review summaries from the time point 1 thoughts and reflections notes, as well as transcripts as appropriate, and incorporate into questions below].

**Introductions**

[Researcher to provide participants with the opportunity to discuss the participant information sheet and privacy sheet, and to ask questions].

**Consent:**

[Researcher to take verbal consent using ‘follow-up’ consent form].

**Preamble:**

*Today is an opportunity for us to explore some more of the information that you provided in previous interviews. We will look again briefly at your social network diagrams and discuss if these have changed in any way. However, the main focus of today will be discussing your local area and how that impacts your levels of physical activity and social connectedness. It does not matter how much physical activity you currently do, nor how socially connected you think you may be. I am interested in how these two things relate to one another in the context of your local area. By physical activity we mean all forms of movement, this could be things like walking, climbing stairs, gardening and housework, or sport and exercise.*

*We will use an online map to look at what facilities and opportunities are available in your area (and help us identifying things missing on the map), then discuss why you go to certain places over others, what you like about your area, and what you think needs to change.*

*There are no right or wrong answers. Please feel free to say as much or as little as you like. You do not have to answer a question if you do not want to. Sometimes, I may ask for a little bit more detail, but if you do not think you have anything else to add, that is fine. Some of the things we talk about today may be sensitive and will be treated in confidence. If you want to take a break or stop the interview at any time just let me know.*

*Are you happy for me to start the recording?*

**Section 1: Changes to physical activity or social connectedness since the previous interview**

[The purpose of this opening section is to ease the participant into the interview and identify whether there are any changes since the previous interview].

How have you been since the last time we spoke in *{insert month}*?

Has there been any changes in your life since that time?

Last time we spoke, the data at the social network interview indicated that you were [less/moderately/highly] active, are your levels of physical activity the same now?

- Probe – what are the reasons for things changing/staying the same?

How do you feel about how active you are currently?

- Probe – would you like to do more or less? Why?
- Probe – (if applicable) what would support you to be more active?

What kind of activities have you been involved in since our last interview? *{consider activities raised during TP1}*

- Probe – what are the reasons for things changing/staying the same?
- Probe – why did you want to try something new?

Last time we spoke, the data collected at the social network interview indicated that you were [never/sometimes/often] lonely, are things the same now?

- Probe – what are the reasons for things changing/staying the same?

**Section 2: Revisiting the social network from timepoint 1**

[The purpose of this section is to identify whether there are any changes to their network since the previous interview].

*As we have discussed, we are now going to revisit the social network diagrams that you help us to create in your first interview. In particular, we are interested in whether there are any changes in your network and if that has impacted your physical activity or social connections.*

[Show network plots and alter list – give participant time to take in the information]

Have there been any changes in your network since these were done?

Are there any new people in your life or people who are no longer involved?

- Probe - What caused these changes?

Are there any people still in your life with whom your relationship has changed?

- Become closer or less close – show plot/data (e.g. concentric rings)
- Relationship has become more positive or negative
- How has the change affected your PA/loneliness?

Have your social groups changed in any way?

- Prompts (*i.e.* broken or newly formed ties within the network)
- Are there any people in your social network that you now particularly rely on for everyday activities (*e.g.* transport/shopping/walking the dog)?
- Probe – you had previously suggested that you rely on *{insert names}* for particular tasks, is this still the case?

Is there anyone in your network who now relies on you for everyday activities?

- Probe – you had previously suggested that *{insert names}* relied on you for particular tasks, is this still the case?

You previously indicated that you were physically active with *{insert names} - a*re you still active with the same people?

- Probe - Is there anybody new that you are now active with? How did that come about?
- Probe – Why are you no longer active with *{insert name(s)}*

Has anything changed in how you socialise with people now that the weather is changing/time of year has changed?

**Section 3: Place**

[The purpose of this section is to explore their local area, its assets, opportunities and accessibility].

*I’d like to know a bit more about your local area. For example, the places you like and dislike, and go and do not go to, and importantly why. I have an online map that we can use to highlight different facilities in your area. We did cover some of these questions in the previous interview, but today we will go a bit more in-depth.*

Before we begin with the online map, can you remind me what your local area is like?

- *Probes – How would you define your neighbourhood? What area does it cover? Is it urban or rural?*
- *Probes - what do you like/dislike about it?*

Where have you been going for physical activity or to meet with others since we last spoke *{insert months}* ago?

- Probes – what prompted this?

Last time we spoke you said that you go to *{insert places}* for physical activity or to meet with others since we last spoke *{insert months}* ago?

- Probes – why is this?

[Show map for first time and explain to participant what it shows, how it works and where they live on the map].

Can you please indicate on this map, how far from your address would you still consider to be local?

- Probe – how far is too far for day-to-day things? (i*.e.* a 20 minute or 30 minute walk)?
- Probe – how far would you consider something to be withing walking distance?
- Probe – what places will you consider walking to?

**Streets and spaces**

*First of all we would like to know what the buildings, streets and public spaces are like in your local area.*

On a scale of 1 to 7, with 1 being the worst and 7 being the best, how would you rate the streets and spaces in your area?

- Probe – why do you say that; what are they like?

Are there any points of interest? [allow participant to point places out on map]

- Prompts – local landmarks, historic features, public squares, parks and gardens, trees
- Probe: Are there interesting and/or beautiful streets, buildings, parks?

Are there any challenges? [allow participant to point out issues on map]

- Prompts – flooding, derelict buildings, vacant land, pollution, litter, lighting, busy roads, pavement clutter, illegal parking, cleanliness and presentation of area
- Prompts – are the streets and spaces around you well maintained?

**Moving around**

*Pleasant and safe routes can encourage people to move around by walking, wheeling and cycling without relying on cars or public transport – this is good for health, and for the environment and air quality. Meanwhile, good public transport is affordable, reliable and well connected. This can reduce reliance on cars and encourage people to get around in ways that are better for the environment and for their health.*

On a scale of 1 to 7, with 1 being the hardest and 7 being the easiest, how easy is it to move around your local area and get to where you want to go?

- Probe – why do you say that?

On a scale of 1 to 7, with 1 being the worst and 7 being the best, how would you rate the public transport in your local area?

- Probe – why do you say that?

Previously you told us that you typically get around your local area by *{insert response from interview 1}*?

- Probe – why is that? Has this changed since we last spoke?

[Use map to show public transport locations]

Is public transport a good option for you?

- Probes - safe and convenient, frequent and reliable, affordable, clean and comfortable, easy to change services
- Probe - Does the public transport system allow you to get to where you need to go if they cannot get there by walking, cycling or wheeling?

Can everyone use the services?

- Probes - all ages and mobility, sensory impairments, mobility aids?

Is there anyone in your social network that you will typically use public transport to meet/travel for social or physical activities?

[Ask participant to point out on the map any paths or routes they like to use for walking and/or cycling]

Are the paths and routes suitable?

- Probe – are there enough routes for people to get to where they want to go?
- Probe – are there any barriers (*e.g.* pavement parking, traffic, overgrown places, bins, lack of dropped curbs, no safe crossing points, no storage for bikes/mobility aids)
- Probe - Can everyone use the routes?

**Feeling safe**

*How safe a place feels can support community activity, affect people’s wellbeing and influence how and where we spend our time. Good design and maintenance can make places feel safe by reducing crime and antisocial behaviour.*

On a scale of 1 to 7, with 1 being the worst and 7 being the best, how would you rate the safety of your local area?

- Probe – why do you say that?

Do you feel safe where you live?

- Probe – why/why not?
- Prompts – consider times of day; demographic differences; dereliction, social issues, disorder and crime; lighting; litter; graffiti; traffic

Can you point out any places on the map that you would feel unsafe or uneasy going [show map]?

- Probe – would you go if you were with somebody else?

**Natural space**

*Good quality natural spaces provide many benefit – improving health and wellbeing, supporting wildlife, reducing flooding, and improving air quality. This includes parks and woodlands, fields, streams, canals and rivers, the coastline, green spaces alongside paths and roads, and tree-lined streets.*

On a scale of 1 to 7, with 1 being the hardest and 7 being the easiest, how easy is it for you to regularly enjoy natural space?

- Probe – why do you say that?

[Use map] This shows the green spaces in your local area *{note different types of green spaces, may also refer to blue spaces}*.

Do you go to any of these places?

- Probe – what are the reasons for this? Do you do physical activity or make social connections there?
- Probe – how often do you go? How do you get there?
- Probe – who in your network/not in your network do you go with/meet there?
- Probe – what do you like or dislike about these places?
- Probe – are these places easy to get to?

Can everyone use the spaces?

- Prompts – disabled people, dog walking, prams and buggies, walking and cycling, playing, local food growing, places to rest?

Thinking about the places you do not go – what stops you using these spaces?

- Prompts – litter, noise, dog fouling, surface mud/dirt, air quality, flooding, no seating, overcrowding, no toilets

How can green spaces in your local area work better for you?

- Probe – provision of activities, type of spaces, use of spaces
- Prompts – drainage, rewilding, protecting and encourage wildlife and nature, tree planting, cleaner air, shade

**Social interactions and physical activity**

*Good places have a mix of spaces and opportunities to do physical activity, and meet and spend time with other people. Some places also have active websites or social media networks to help people meet and take part in the local community. Being inactive or feeling isolated can be damaging to our health and wellbeing.*

[Using each of these map layers in turn (‘leisure’, ‘places to eat and drink’ and ‘shopping and retail’), ask the following questions]

On a scale of 1 to 7, with 1 being the worst and 7 being the best, how would you rate the range of opportunities that allow you to meet and spend time with other people in your local area?

- Probe – why do you say that?

On a scale of 1 to 7, with 1 being the worst and 7 being the best, how would you rate the spaces and opportunities for physical activity in your local area?

- Probe – why do you say that?

[Show map] This shows all of the locations for *{insert layer}* in your local area:

Do you go to any of these places?

- Probe – what are the reasons for this? Do you do physical activity or make social connections there?
- Probe – how often do you go?
- Probe – who in your network/not in your network do you go with/meet there?
- Probe – what do you like or dislike about these places?

Why do you not go to/use *{insert places}* for physical activity/social connections?

- Probe – issues such as access, location, lighting, noise, cost, public toilets, traffic, weather, activities not welcomed by the community

Can you show me on the map where else you go to get together with other people/do physical activity?

- Probe – can everyone join in and mix (*i.e.* accessible, inclusive, welcoming, free or affordable, digital access and skills)?

*Ask participants about whether they feel schools, or places for religion and worship, are relevant to go through on the map – if so, then ask:*

- *In what ways do they influence your PA or social interactions?*
- *What is the role of churches/schools in your local area?*
  - *Prompt – range of facilities and activities*

[Stop using map]

**Neighbourhood**

Do people know each other/do neighbours interact in your local area?

- *Is there a sense of community?*
- *Do people in your local area help one another? (e.g.* elderly neighbours)

**General**

*Thinking about opportunities that allow people to meet and spend time with other people socially or to be physically active.*

- Are there any gaps? How could we make things better in future? How else can we make the most of what your local area has to support i) physical activity; and ii) social connectedness?
- Probe – type of activities, type of spaces, use of spaces

Is there anything else you would like to add that we have not covered?

**Debrief**

[Provide opportunity for participants to offer final remarks and ask questions. Outline the next steps, explain data use and remind about voucher].
